# Radiological changes do not influence clinical mid-term outcome in stemless humeral head replacements with hollow screw fixation: a prospective radiological and clinical evaluation

**DOI:** 10.1186/s12891-018-1945-6

**Published:** 2018-01-22

**Authors:** Philipp R. Heuberer, Georg Brandl, Leo Pauzenberger, Brenda Laky, Bernhard Kriegleder, Werner Anderl

**Affiliations:** 1 St. Vincent Shoulder and Sports Clinic Vienna, Baumgasse 20A, 1030 Vienna, Austria; 2 Austrian Research group for Regenerative and Orthopedic Medicine (AURROM), Vienna, Austria

**Keywords:** Stemless shoulder arthroplasty, Mid-term outcome, Radiological and clinical results, Hemiarthroplasty, Anatomic shoulder arthroplasty

## Abstract

**Background:**

Stemless shoulder arthroplasty is a fairly new concept. Clinical and radiological follow-up is essential to prove implant safety and concept. This prospective single-centre study was performed to evaluate the influence of radiological changes on clinical mid-term outcome following stemless humeral head replacement with hollow screw fixation.

**Methods:**

Short- and mid-term radiological and clinical evaluations were performed in 73 consecutive shoulders treated mainly for idiopathic and posttraumatic osteoarthritis with stemless humeral head arthroplasty including 40 hemi- (HSA) and 33 total shoulder arthroplasties (TSA). Operating times of stemless implantations were compared to 110 stemmed anatomical shoulder prostheses. Appearances of humeral radiolucencies or radiological signs of osteolysis or stress shielding were assessed on standardized radiographs. Patients’ clinical outcome was evaluated using the Constant score and patients’ satisfaction was documented.

**Results:**

Radiological changes, detected in 37.0%, did not affect clinical outcome. Constant scores significantly improved from baseline to short and mid-term follow-up (*p* < 0.001). The majority of patients (96.2%) were satisfied with the procedure. No loosening of the humeral head component was detected during a mean follow-up of 58 months. Operating times were significantly shorter with stemless compared to stemmed implants (*p* < 0.001).

**Conclusions:**

Clinical mid-term outcome after stemless humeral head replacement was not affected by radiological changes.

**Trial registration:**

The institutional review board (St. Vincent Hospital Vienna; 201212_EK01; date of issue: 11.12.2012) approved the study. The trial was registered at ClinicalTrials.gov (NCT02754024). Retrospective registration.

## Background

**S**houlder arthroplasty is the standard treatment for patients with severe shoulder osteoarthritis with numbers continually rising. Modern anatomically designed shoulder arthroplasty systems have proven to provide significant pain relief and improvement of function over time [1]. Long-term survivorship is mostly affected by implant related complications [2]. Although loosening of the glenoid component is more common than stem related problems [3], given the growing number of shoulder replacements and revision surgeries, the humeral side becomes increasingly clinically relevant. Loosening is not the only indication for revision, as removal of the humeral stem is also required for implant unrelated complications such as infection or secondary rotator cuff tear. In revision surgery, complications commonly arise with difficulty of stem removal, cement extraction, bone loss or osteolysis due to polyethylene wear debris, and intraoperative fracture associated with potentially longer operating times, increased risk of infection, nerve injuries, and worse outcome [1, 4–6]. Shaft malunion after fracture, where the deformity does not allow an anatomic positioning of the humeral stem [7, 8], is another challenging situation to treat.

An expedient alternative is humeral head resurfacing. Nevertheless, humeral resurfacing is a technically challenging procedure requiring at least 60% of intact humeral head [9]. Firstly, it is mandatory to find the correct centre of rotation to avoid overstuffing and secondly with the remaining head in situ correct glenoid component implantation may be difficult [10].

A contemporary, promising alternative are stemless anatomic shoulder implants, which generally rely on metaphyseal or meta- and epiphyseal fixation, combining the advantages of humeral head resurfacing and common shoulder replacement. Potential benefits are (1) the possibility of anatomic shoulder reconstruction regardless of medial and posterior offset of the proximal humerus, caput-collum-diaphyseal angle, and retrotorsion of the humeral head; (2) the ability to perform implantation in fracture sequelae with shaft malunion and secondary post-fracture situations with humeral head deformities; (3) good glenoid exposure; (4) bone stock preservation; and (5) facilitating further revision surgery.

The main purpose of this prospective study was to evaluate radiological changes around the humeral component of a stemless shoulder implant with hollow screw fixation and their influence on clinical outcome. We hypothesized that clinical mid-term outcome after stemless humeral head replacement would be affected by radiological changes. Secondary objectives were to compare operating times between stemless and stemmed hemi- and total shoulder arthroplasty and to report short and mid-term clinical outcome after stemless hemi- and total shoulder arthroplasty.

## Methods

**Fig. 1:**
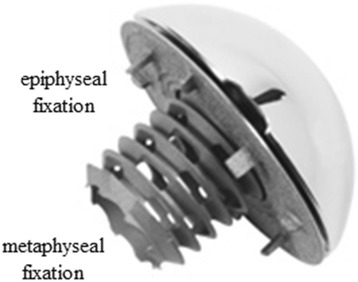
The stemless humeral head Eclipse prosthesis consists of three components: the anatomical humeral head; the trunion for epiphyseal fixation; and the hollow cage screw for metaphyseal fixation. Published with permission from (Arthrex Inc., Naples, FL, USA)

Between September 2005 and December 2011, a total of 136 shoulder arthroplasties using a stemless humeral head implant were performed by two experienced surgeons (W.A., B.K.) at our clinic. The stemless humeral head replacement (Eclipse™ Shoulder Prosthesis, Arthrex Inc., Naples, FL, USA) consists of an anatomical humeral head, which is mounted on a baseplate (trunion) for cortical support at the anatomical neck (epiphyseal fixation) and fixated to the cancellous metaphyseal bone over a screw-in mechanism with a hollow screw (Fig. 1).

Indications for surgery included primary or secondary osteoarthritis of the shoulder with severe pain and limited function. Patients with any kind of rotator cuff tears, cuff tear arthropathies, axillary nerve lesions, or revision arthroplasty were not suitable for this procedure. HSA was indicated, when the glenoid presented without erosion and macroscopically fairly intact cartilage. Included were all patients between 40 and 85 years of age at the time of surgery, who were scheduled for primary anatomical shoulder replacement for the treatment of degenerative, rheumatic, posttraumatic osteoarthritis and fracture sequelae. Institutional review board approval (201212_EK01) was obtained. The trial is registered at ClinicalTrials.gov (NCT02754024).

A total of 95 eligible patients with 100 stemless humeral head implants signed the written informed consent and were enrolled in the prospective trial. Patients who died or withdrew before the mid-term follow-up were excluded. During the study period 11 patients died from unrelated causes (five before and six after the short follow-up) and one patient with bilateral implants withdrew from the study before the short follow-up due to an unexpected comorbidity. A complete, prospectively collected data set before, two years (short follow-up) and at least 48 months (mid-term follow-up) after surgery, or until indicated revision surgery was required for inclusion of radiographic and clinical evaluation.

Additionally, operating times of a consecutive cohort of 93 patients (100 implants), who underwent anatomical shoulder arthroplasty with a stemmed anatomical shaft prostheses (Anatomical Shoulder™ System, Zimmer Inc., Warsaw, IN, USA) by the same two experienced surgeons (W.A., B.K.) at our clinic were obtain.

### Surgical procedure

Patients were operated under general anaesthesia combined with an interscalene block. Through a standard deltopectoral approach after tenotomy of the subscapularis tendon and exposition of the humeral head, a resection at the anatomical neck was performed.

Thereafter, for the stemless procedure, the humeral head size was determined using templates and the best fitting length of a hollow screw, which allows trabecular ingrowth into the bone and fixation to the trunion, was assessed with a measure pin. A hole for the cage screw was prepared with a hand coring reamer. In case of glenoid replacement, a standard cemented all-polyethylene glenoid component (Arthrex Inc., Naples, FL, USA) was implanted. Again the humeral head was exposed and with a centering device, the trunion of the previously determined head size was impacted for epiphyseal fixation. Thereafter, the appropriate hollow screw was advanced until the screw head was flush with the neck of the trunion. Finally, the correct humeral head was mounted. The subscapularis tendon was reattached with two suture anchors (titanium Corkscrew FT-III, Arthrex) and the wound was closed. Immediately postoperative, fluoroscopic images in two planes were taken to confirm correct implant positioning. Routine follow-ups were scheduled two weeks, three months, one, two, and five years after the procedure. The operating time from incision to final closure for each shoulder replacement was prospectively recorded.

Postoperatively, the arm was secured with an abduction cushion (UltraSling, Breg MDSS GmbH, Hannover, Germany). The physiotherapy regimen included immediate passive motion and passive assisted motion with closed chain exercises to work scapula-thoracic muscles after up to seven days. After six weeks patients were allowed to remove sling; active movements and strengthening exercises started six weeks after implantation.

### Clinical evaluation

Pre- and postoperative clinical and functional investigations included the Constant Score [11] adjusted for age and gender [12]. All clinical evaluations were performed by orthopaedic fellows. A single subjective question categorized into very satisfied, satisfied, and unsatisfied was asked to evaluate patient satisfaction. Complications and necessity of revision surgery were recorded. Clinical outcome before revision surgery was recorded, but not included in short- and mid-term analyses.

### Radiographic evaluation

Pre-, peri-, two- and five-year postoperative radiographs of the humeral head in anteroposterior and axial view using a fully-digital 2-in-1 system (AXIOM Luminos dRF, Siemens, Munich, Germany) were assessed by one experienced radiologists (rater 2) and two experienced orthopaedic surgeons (rater 1 and 2). Differences in radiological ratings were discussed to find a consensus. In case no consensus could be reached, the final decision was made by the senior author (rater 1). This grading was used for data analysis.

**Fig. 2:**
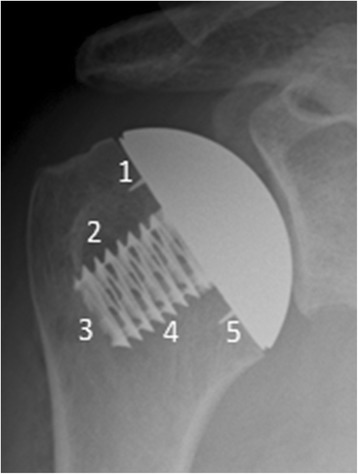
Anteroposterior radiographs of the stemless humeral head implant showing five radiographic zones including the superior and inferior interface (zone 1 and 5) and the interfaces around the hollow screw (zones 2, 3, and 4), which were used for the assessment of radiolucency and osteolysis

In the event of revision surgery, radiographs obtained before the second procedure were evaluated. Presence of or progression of radiolucency, especially around the bone-implant interface, was recorded. Five radiographic zones including the superior and inferior interface (zone 1 and 5) and the interface around the hollow screw (zones 2, 3, and 4) were assessed for radiolucent areas or osteolysis (Fig. 2).

A radiologic classification system for this specific implant was established using allocations for radiolucent lines similar to Cil et al. [13], and for osteolysis and internal stress shielding similar to Raiss et al. [14]. Radiological findings were grouped into three groups: (group1) no lucency or osteolysis with minor radiolucent lines in one or more zones; (group2) minor radiological findings including major radiolucent lines or partial osteolysis in one or more zones; and (group3) major radiological findings and complete osteolysis in one or more zones. Humeral head implants were considered to be at risk of loosening, if complete osteolysis was detectable in two or more zones. Possible internal stress shielding (loss in bone density around the hollow screw) was also described.

### Statistical analysis

For sample size calculation, we assumed that 20% of implants will present with radiological changes and that postoperative Constant scores of those patients will be approximately 70 (60–80) %. Thus, we assume that those patients without radiological changes (80% of patients) will have significantly better Constant scores (approximately 80 (70–90) %). If the true difference in mean Constant scores is 10% (independent *t*-test), we planned to study 10 patients with and 42 without radiological changes to be able to reject the null hypothesis that the population means of the two groups are equal with probability (power) 0.8. The Type I error probability associated with this test of this null hypothesis is 0.05. To account for exclusions and loss to follow-up we enrolled 100 cases.

Descriptive statistic was used to present patient characteristics. Data distribution was assessed by a visual inspection of histograms and the Kolmogorov-Smirnov-test. Categorical data were assessed using Fisher’s exact tests. Differences regarding age- and gender-adjusted Constant scores as well as operating times between two independent groups were calculated using independent t-tests. Paired t-tests were used to compare age- and gender-adjusted Constant scores between pre- and postoperative assessments. Kappa statistic was performed to determine consistency regarding radiographic gradings among raters. All data were analysed using SPSS software (PAWS Statistics 21; SPSS Inc., Chicago, IL). Statistical significance was set at a *p*-value of < 0.05 (two-sided).

## Results

Of the 83 patients (87 shoulders) who met inclusion criteria, 11 patients (three with bilateral implants) were lost to follow-up or had incomplete data (follow-up rate: 86.7%). Demographics of 72 patients (73 shoulders; Table 1) were comparable between stemless HSA (*n* = 40) and TSA (*n* = 33) regarding age (68.0 ± 10.1 range, 44–84; vs. 67.2 ± 10.5 range, 40–83; *p* = 0.752); gender (female: 75.0% vs. 54.5%, *p* = 0.085); and indication (idiopathic osteoarthritis: 75.0% vs. 87.9%, posttraumatic osteoarthritis: 15.0% vs. 9.1%, humeral head necrosis: 7.5% vs. 3.0%, and instability arthritis: 2.5% vs. 0%, respectively; *p* = 0.500).

**Table 1:** Patient demographics

	Stemless humeral head replacement (*n* = 73)
Gender (n)	48 female (65.8%) / 24 male (1 bilateral; 34.2%)
Mean age at surgery (years)	67.6 (SD 10.2; range, 40–84)
Indications	
idiopathic osteoarthritis (n)	59 (80.8%)
posttraumatic osteoarthritis (n)	9 (12.3%)
humeral head necrosis (n)	4 (5.5%)
instability arthritis (n)	1 (1.4%)

*Abbreviation*: *HSA* hemi shoulder arthroplasty, *SD* standard deviation, *TSA* total shoulder arthroplasty

A comparison of the duration of the surgical procedure between stemless and stemmed HSA as well as TSA showed significantly shorter operating times for stemless shoulder arthroplasty (Table 2).

**Table 2:** Comparison of operating times between stemless and stemmed shoulder arthroplasties

Operating Time^a^ (minutes)	Stemless	Stemmed	*p*-value
HSA	73.2 (SD, 17.8; range, 42–129)	95.1 (SD, 23.0; range, 35–143)	< 0.001
TSA	95.7 (SD, 20.3; range, 67–163)	120.7 (SD, 36.4; range, 53–249)	< 0.001

*Abbreviation*: *HSA* Hemi Shoulder Arthroplasty, *TSA* Total Shoulder Arthroplasty

^a^The values are given as the mean and standard deviation and range in parenthesis

Postoperative clinical assessment was performed after an average of 27.7 (22 to 37) months (short follow-up; *n* = 53) and 58.1 (48 to 87) months (mid-term follow-up; *n* = 53). Radiographs of patients with an indication for revision surgery (*n* = 7) or with revision surgery (*n* = 13) were assessed 38.3 (4 to 99) months postoperatively. The reasons for revision surgery and those with an indication for a further procedure are listed in Table 3. Revision surgery was mainly indicated for secondary glenoid erosion in patients with HSA (61.5%) and secondary rotator cuff tendon tear for patients with TSA (71.4%). Not significant, but more patients with HSA (32.5%) compared with TSA (21.2%) were revised (*p* = 0.306). None of the patients required revision surgery for humeral implant loosening. Patients not requiring revision surgery showed no signs of glenoid erosion (HSA), stable glenoid components (TSA), and intact rotator cuff situations at final follow-up.

**Table 3:** Reasons for Revision Surgery

	HSA (*n* = 13)	TSA (*n* = 7)
Implant loosening of the humeral head component	0	0
Secondary glenoid erosion or	8	n.a.
Loosening of the glenoid component	n.a.	0
Secondary rotator cuff tendon tear (SSP or SSC)	4	5
Postoperative SNOH fracture	0	1
others	1	1

*Abbreviation*: *HSA* Hemi Shoulder Arthroplasty, *n.a.* not applicable, *TSA* Total Shoulder Arthroplasty, *SNOH* surgical neck of humeral, *SSC* Subscapularis, *SSP* Supraspinatus

**Fig. 3:**
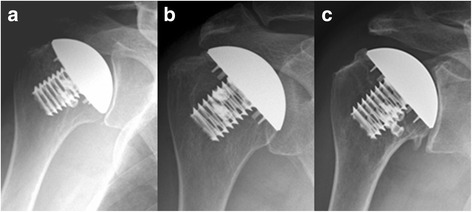
Anteroposterior radiographs of a seventy-two-year-old woman made immediately after hemi shoulder arthroplasty (**a**), at the short-term follow-up showing no radiolucency or osteolysis (**b**), and at the mid-term follow-up showing osteophyte formation superior around the humeral head implant and inferior (**c**)

**Fig. 4:**
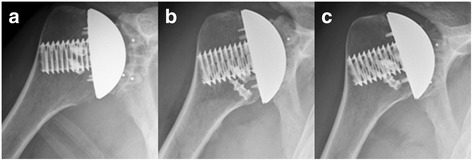
Anteroposterior radiographs of a forty-year-old man made immediately after total shoulder arthroplasty (**a**), at the short-term showing partial osteolysis at the inferior trunion bone interface (**b**), and at the mid-term follow-up showing stable partial inferior osteolysis and minor superior radiolucency (**c**)

Rater agreements for radiologic gradings were very good (rater 1 and 2: κ = 0.871, 95% confidence interval (CI): 0.764–0.978; rater 1 and 3: κ = 0.920, 95% CI: 0.833–1.000; rater 2 and 3: κ = 0.898, 95% CI: 0.802–0.994). While humeral implant loosening was not the reason for any revision surgery, according to radiological findings, 11.0% (8 of 73) of humeral head implants were found to be at risk of loosening considering complete osteolysis in two or more zones. Only three of the 12 revised implants were radiologically at risk of humeral head loosening. However, radiological loosening could not be confirmed intraoperatively in those three TSA cases, which were revised for secondary rotator cuff tears. All other implants showed an uneventful progress over time (Figs. 3 and 4).

Radiological changes (group 2 and 3) were detected in 37.0% (HSA: 17.8%; TSA: 19.2%). Partial osteolysis or major radiolucencies (group 2) were detected in 4.1% with HSA and in 2.7% with TSA, while complete osteolysis in one or more zones (group 3) was present in 13.7% in the HSA and 16.4% in the TSA-group (Table 3).

Patients with revision surgery did not have significantly more complete osteolysis in one or more zones than those without revision surgery (30.0% vs. 30.2%, *p* = 1.000; HSA: 15.4% vs. 29.6%, *p* = 0.451; TSA: 57.1% vs. 30.8%, *p* = 0.377, respectively). Internal stress shielding, defined as reduced bone mineral density around the hollow screw, was seen in 42.5% of patients. The number of implants showing internal stress shielding did not differ between patients with and without a revision procedure (25.0% vs. 47.2%, *p* = 0.112; HSA: 30.8% vs. 48.1%, *p* = 0.333; TSA: 14.3% vs. 46.2%, *p* = 0.202, respectively). Further details regarding radiological outcome are provided in Table 4.

**Table 4:** Radiological Outcome with Stemless Humeral Head Replacement at Final Follow-Up

	HSA (*n* = 27)	HSA REV (*n* = 13)	TSA (*n* = 26)	TSA REV (*n* = 7)	TOTAL (*n* = 73)
Group 1	18	9 (4 s OA + 4 s RCT + 1 undefined pain)	16	3 (1 s RCT + 1 HH Fx + 1 undefined pain)	46
no RL/OL or minor RL in one or more zones					
No radiolucency	16	7	12	3	38
Zone 1 (minor RL)	1	2	1	0	4
Zone 5 (minor RL)	1	0	2	0	3
Zone 1 (minor RL) + Zone 5 (minor RL)	0	0	1	0	1
Group 2	1	2 (2 s OA)	2	0	5
major RL or partial OL in one or more zones					
Zone 1 (major RL)	1	1	2	0	4
Zone 1 (partial OL) + Zone 5 (partial OL)	0	1	0	0	1
Group 3	8	2 (2 s OA)	8	4 (4 s RCT)	22
complete OL in one or more zones					
Zone 1 (major OL)	0	0	1	0	1
Zone 5 (major OL)	4	1	5	1	11
Zone 1(minorRL) + Zone 5 (majorOL)	2	0	0	0	2
Zone 1(majorOL) + Zone 5 (majorOL)	2	1	2	3	8
No internal stress shielding	13	9 (5 s OA + 3 s RCT + 1 undefined pain)	14	6 (5 s RCT + 1 HH Fx)	42
Internal stress shielding in one zone (1, 2, or 4)	8	2 (2 s OA)	5	0	15
Internal stress shielding in two zones (12, 23, 24, or 34)	5	2 (1 s OA + 1 s RCT)	6	1 (1 undefined pain)	14
Internal stress shielding in three zones (125 or 234)	1	0	1	0	2

*Abbreviation*: *HH Fx* Humeral Head Fracture, *HSA* Hemi Shoulder Arthroplasty, *OL* osteolysis, *sec OA* secondary Osteoarthritis, *REV* revision surgery, *RL* radiolucent line, *sec RCT* secondary Rotator Cuff Tear, *TSA* Total Shoulder Arthroplasty

Clinical outcome was not significantly affected by radiological changes (Table 5).

**Table 5:** Clinical mid-term outcome of 53 stemless humeral head replacements compared to radiological findings

Radiological findings	N	Constant score (%)	*p*-value
No or minor radiological findings	37	78.9 (SD, 15.7; range, 30.5–101.2)	0.891
Major radiological findings	16	78.3 (SD, 15.8; range, 49.4–97.5)	
No internal stress shielding	28	80.4 (SD, 15.6; range, 49.4–101.2)	0.411
Internal stress shielding in one or more zones	25	76.8 (SD, 15.7; range, 30.5–98.8)	

**Fig. 5:**
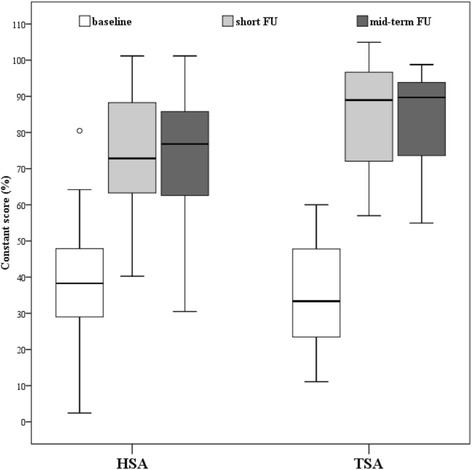
Clinical outcome between hemi- (HSA; *n* = 27) and total shoulder arthroplasty (TSA; *n* = 26) at baseline (HSA: 38.8 ± 17.0% vs. TSA: 36.6 ± 13.9%, *p* = 0.594), at short (HSA: 74.0 ± 16.5% vs. TSA: 83.9 ± 14.4%, *p* = 0.024) and mid-term follow-up (HSA: 73.5 ± 17.3% vs. TSA: 84.2 ± 11.6%, *p* = 0.011)

Clinical outcome has significantly improved from pre- to both postoperative follow-ups (37.7 ± 15.4% range, 2.4–80.5 vs. 78.8 ± 16.1% range, 40.2–104.9; *p* < 0.001; and vs. 78.7 ± 15.6% range, 30.5–101.2; *p* < 0.001), but no significant changes (*p* = 0.947) were detectable between the follow-ups (Fig. 5). TSA showed significantly better clinical outcome than HSA at short and mid-term follow-up (Fig. 5). Furthermore, at the mid-term follow-up 96.2% of the patients were very satisfied (15 HSA, 20 TSA) or satisfied (10 HSA, 6 TSA).

## Discussion

Previous studies have been reporting radiological and clinical findings after stemless anatomical shoulder arthroplasty; however, to our knowledge we are the first to compare radiological findings to clinical mid-term outcome in a prospective single-centre trial. We were not only able to show that clinical and radiological results with the stemless shoulder prosthesis showed favourable improvements from pre- to short and mid-term outcome with no revision surgery due to humeral component loosening, we also had to reject our hypothesis that radiological changes would negatively impact clinical outcome. Constant scores were not significantly different between patients with and without radiological changes in the mid-term follow-up.

Recently, comparable Constant scores were detected by Habermeyer et al. [15]. In accordance to the literature for stemmed shoulder arthroplasty, TSA showed significantly higher relative Constant Scores than HSA in this study, and values remained stable over time [16, 17]. As the present study and current literature [18, 19] demonstrated, stemless shoulder arthroplasty provides similar clinical results as established stemmed arthroplasty systems. The main focus of our study was the radiologic behaviour of the implant and its influence on clinical outcome. Interestingly, the present results regarding radiolucencies and osteolysis are rather different to the findings of Habermeyer et al. [15], who reported an osteolysis rate of not more than 4%. Such variations could be explained by the fact that radiographs were assessed and classified differently. While we adapted a rather concise nomenclature, Habermeyer did not provide detailed information regarding the classification protocol [15]. Nevertheless, the presence of osteolysis did not affect clinical outcome or revision rates. Comparable to Habermeyer’s findings [15], internal stress shielding occurred in half of the implants in one or more zones with the majority around the greater tuberosity. Again, no influence on clinical outcome could be seen in either study.

The majority of abnormal radiological findings occurred either in the superior part of the trunion or in the inferior implant bone interface near the calcar, often leading to partial osteolysis similar to the already known phenomenon described by Raiss [14]. Radiolucencies in the superior and inferior parts of the trunion could be explained with the results of finite element analyses (FEA) [20]. FEA suggested that superposition of force and moment induced stresses in weak inferior cortical bone support is leading to higher migration values within the inferior trunion-bone interface and is therefore resulting in a shift of the centre of rotation. The bone adaptation and resorption around the implant is mostly dependent on the stability of the hollow screw and is caused by axial bending stiffness similar to the concept explained by Nagels et al. [21]. Since such radiological findings were not reflected in the clinical outcome, an arbitrary definition of an implant at risk had to be established. Thus, we assumed that if an implant showed the presence of osteolysis in two or more zones, the humeral head component might become unstable.

Currently, few different stemless shoulder implant systems are on the market with sporadic report of short-term outcome. A major strengths of the Eclipse prosthesis is that it is the only implant with a different fixation mechanism not relying on impaction implantation and therefore avoiding a potential risk of metaphyseal fracture or implant migration [19].

Revision surgeries followed an already well-known pattern. In hemiarthroplasties the majority of revisions were due to secondary glenoid erosion (20.5%), which was similar to other studies [22]. Secondary cuff failure was the main cause for revision (15.2%) in total shoulder arthroplasty. The higher revision rate due to secondary rotator cuff ruptures in the present trial compared to rates (< 8%) reported by Sperling et al. [23] could be explained with the relatively high mean age in the TSA-group. Other than that, reasons for revision were traumatic surgical neck of humerus fracture, undefined pain for neglected long head of biceps tendon, and symptomatic mes acromion. However, no revision surgery was indicated due to humeral component loosening or polyethylene glenoid component loosening in this patient cohort.

Fortunately, but not surprisingly, revision surgery with such a stemless implant compared to stemmed prostheses were fairly easy as the removal of the humeral component does not require any special manoeuvres (e.g. osteotomy) and leaves a rather anatomic situation with the whole metaphysis in place.

Last but not least, shorter operating times, another strength of stemless humeral head surgery, with HSA as well as with TSA in comparison with a stemmed anatomic shoulder arthroplasty system could be observed. This fact is not surprising, but worth mentioning since 20 min less on operating time is a benefit for the patient regarding reduced anaesthesia time and blood loss, not to mention the advantage in time management and therefore cost effectiveness. As the humeral component implantation is a rather easy procedure, which is also reflected by the significant shorter operating time compared to stemmed shoulder arthroplasty, the learning curve for this surgical technique is quite steep.

Although results of this mid-term trial are promising, long-term outcome regarding implant stability needs to be reported. Another limitation is that in-depth subjective assessments (e.g. American Shoulder and Elbow Surgeons or quality of life scores) are missing. Furthermore, assessment of bone mineral density in the humeral head prior to implantation would have been desirable as bone mineral density might influence implant ingrowth and the possible appearance of osteolysis [24]. Unfortunately, our institution does not offer the possibility to assess bone mineral density in the shoulder. However, we believe that the present results outweigh these limitations by the thorough radiographic analysis demonstrating that radiological findings do not influence clinical outcome. We speculate that in the future a humeral head replacement with a shaft may only be indicated under rare circumstances, while the majority of patients, at least according to our results, can be successfully treated with a stemless humeral head replacement.

## Conclusion

Promising clinical mid-term results with or without minor radiographic findings suggest that the concept of this stemless humeral head prosthesis might be an expedient alternative to existing shoulder replacements. Anatomical cutting of the humeral head and hollow screw fixation allow a familiar access to the glenoid combined with a shaft independent, less complex bone-sparing surgery along with shorter operating times and favourable revision situation.

## Abbreviations

FEA: Finite element analyses
HSA: Hemi shoulder arthroplasties
TSA: Total shoulder arthroplasties (TSA)
